# The evolving landscape of snakebite policies in India

**DOI:** 10.1371/journal.pntd.0013145

**Published:** 2025-07-09

**Authors:** Akhilesh Kumar, Maya Gopalakrishnan

**Affiliations:** 1 Department of Medicine, Lady Hardinge Medical College, New Delhi, India; 2 Department of Medicine, All India Institute of Medical Sciences, Jodhpur, India; Universidad de Costa Rica, COSTA RICA

To the Editor,

In the article ‘From neglect to equity in snakebite envenoming; what the ICMR-Collaborative Centre of Excellence (CCoE) targets’ published on 12^th^ September 2024, Menon et al., acknowledge the challenges ahead in addressing snakebite envenomation in India, while identifying the high-priority areas for improvements such as access to antivenom, venom research, healthcare delivery and policies [1]. What begins as an acute problem has the potential to cause death, long-term morbidities with huge financial strain on the family ruining their livelihoods and resulting in long-lasting psychological issues. Hence, snakebite envenomation is not just a medical emergency, it is a social hazard perpetuated due to socioeconomic inequalities and poverty, affecting communities, that have limited resources and are in rural areas.

Efforts to mitigate snakebite envenomation in India have gained more visibility and traction with the declaration to launch national programme for the prevention and control of snakebite envenomation in September 2022 [2]. India is the first country to have a national programme against snakebite envenomation. Consequently, the National Action Plan for Prevention and Control of Snakebite Envenoming (NAPSE) was launched by the Ministry of Health and Family Welfare (MoHFW) on 12^th^ March 2024 with the aim to halve snakebite deaths by 2030 through a ‘One Health’ approach [3]. To strengthen the surveillance systems, snakebite envenomation has been made a notifiable disease by MoHFW on 27^th^ November 2024 [4]. More recently, the Supreme Court of India took cognizance of the snakebite problem and has called for responses from the Centre and states regarding measures to improve access to anti-venom and snakebite treatments throughout India [5]. As India contributes the most to the global snakebite burden, these efforts showcase the much-needed momentum around snakebite mitigation efforts in India. However, the NAPSE also carries the baggage of disadvantages inherent to a single condition-focused vertical healthcare program, such as fragmentation of services, resource allocation issues, challenges with equity, and integrating care at the primary care level while also being costly [6]. Furthermore, snakebite envenoming is unique among neglected tropical diseases (NTDs) because it cannot be prevented or addressed through quick-fix measures such as vaccination or mass drug administration, where a top-down approach with donor-mediated funding can be implemented and easily audited [7]. Instead, addressing the snakebite challenge needs fundamental health system reforms from a reactionary, demand-based care to a prevention focused universal healthcare at the primary level.

As Menon et al, have rightly pointed out “Health” in India is currently under the State List (Seventh Schedule of the Constitution of India); the responsibility to implement healthcare policies, irrespective of the subject lies with individual state governments [1]. In a country as huge as India with >60 venomous snake species, the impact of envenomation including circumstances when bites happen, seasonal variations, diverse socio-cultural milieu, issues with healthcare access, the role of traditional healers, heterogenous clinical manifestations, and long-term morbidity varies from region to region [8,9]. Hence a regionally tailored collaborative approach with different stakeholders (vulnerable communities, researchers, health practitioners, industries, non-governmental organization, and policymakers) within various regions is warranted for an effective implementation of the NAPSE. All the important measures mandated by the NAPSE including - the assurance of adequate and uninterrupted antivenom supply, improving capacity building & strengthening healthcare services, accessibility, regional antivenom centres, intersectoral collaboration, surveillance, and notifying snakebites require a thorough understanding of local issues, which can be achieved only locally [4]. A single decision-making body without clarity on how year-on-year funding will be allocated to the states may not help in the impactful implementation of many of the proposed policies of NAPSE.

The timely availability of appropriate antivenom is the most important action critical for snakebite management, hence, primary and community health centres must remain the key focus for any policy implementation. However, evaluation of healthcare structural capacity across different states of India reveals gross deficiencies and a need for comprehensive improvement of India’s primary healthcare system for optimal snakebite management [10]. Strengthening the healthcare system, capacity building of healthcare workers, implementing cutting-edge technologies, uninterrupted funding support, and most importantly - community engagement and community development are needed for effective preventive measures, improving access, and optimizing health service use. Hence, the interventions should be planned in a decentralised manner addressing the varying needs of each place.

Decentralised health programmes have been effective in many low- and middle-income countries [11]. For example, one vertical program which has remained successful is the National Tuberculosis Elimination Programme (NTEP) implemented under the National Health Mission, a collaboration between the Indian Union Government and state governments. National Tuberculosis Elimination Programme had decentralised tuberculosis services with State Tuberculosis Training and Demonstration Centre (STDC) and cross-sector partnerships aiming to strengthen health response and the programme helped in a 17.7% reduction in incidence from 2015 to 2023 [12]. State Tuberculosis Training and Demonstration Centres are involved with the planning and execution of need-based Operational/ Implementation Research [13]. The Road Map for Neglected Tropical Disease 2021–2030 has highlighted the need for a cross-cutting approach [14]. It is possible to go further with snakebite envenoming by integrating it with other health programs such as unifying post-bite wound and limb care with existing provisions for lymphatic filariasis, leprosy, and diabetic foot care at the primary level.

In terms of research priorities, the gap areas are: basic science research into snake species mapping, evolutionary genomics, venomics and antivenomics, epidemiological studies to determine burden, clinical studies to delineate pathophysiology, and qualitative studies to better understand the stigma and neglect faced by the vulnerable communities. Further, intervention research for new and improved antivenoms and crucially, implementation and operational research to gain feedback on success of the policy are needed. While the creation of a single Centre of Excellence (CoE) appears to be a welcome step, placing it in the high echelons of the few centres worldwide that are currently devoted to snakebite research, we must pause to reflect and examine, whether it is the best way forward as a strategy for promoting and supporting snakebite research in India.

For the long list of mandates which the Collaborative Centre of Excellence (CCoE) wishes to fulfil, it appears that project merit- based funding is a grossly inadequate mechanism to achieve these goals. For example, the current polyvalent antivenom available in India is effective only against the ‘Big Four’ snakes however, regional venom variations and challenges with antivenom inefficacy among the ‘Big Four’ are reported [15–17]. The Big Four refers to Russell’s viper (*Daboia russelii*), Saw-scaled viper (*Echis carinatus*), Common krait (*Bungarus caeruleus*), and Indian cobra (*Naja naja*), responsible for 90% of snakebite deaths in India. Apart from this, there are regional species against which no antivenom is available. While zonal venom pools at a CCoE appear to be a good first step to counter this, regional antivenom production with the involvement of various state governments will be needed to help ease the availability of effective antivenoms. However, it is unclear how the CCoE will facilitate the translation of basic science research to effective regional antivenoms given the centre-state disconnect and a project-based funding approach.

The authors mention that the national study completed by CCoE covered 14 out of 28 states and 8 union territories of India with 2–4 districts from each state. The efforts for this massive and complex exercise are commendable, especially since it has never been undertaken before. However, with 787 districts in India, we still have a long way to go before achieving a truly nationally representative picture. This limitation is likely due to challenges in logistics and insufficient funding support in the top-down approach. A better solution is to evolve a bottom-up decentralised approach with robust reporting services rooted in the community. Though snakebite has been made a notifiable disease with reporting from healthcare facilities, under-reporting can occur with cases not accessing healthcare services. However, being a traumatic event (less chance of recall bias), snakebite can be easily reported at the community level by health workers with the help of local leaders and traditional healers (similar to pregnancy and infant care service delivery). While it is important to have knowledge partners for evidence generation, CCoEs may end up restricting access to research support and funding. It is crucial to provide support to the entire community of snakebite researchers - clinical and non-clinical, institutional or otherwise including implementation research and, to address the wide array of issues in different regions. Decentralisation of funds, availability of resources, and involvement of local institutions to gain access to platforms for research will reduce the existing gap and help in snakebite mitigation. Similarly, collaboration between different stakeholders in various areas or states is warranted for effective intervention at the national level. Therefore, future policies must be suited to meet these complex challenges making efforts to shift the balance towards patients, families, and their local representatives by meticulous planning for the implementation of NAPSE while integrating it with other health program delivery.
